# The Atlanta Medical College Commencement

**Published:** 1886-04

**Authors:** 


### THE ATLANTA MEDICAL COLLEGE COMMENCEMENT.

The commencement exercises of this college were held in DeGive's Opera House on the evening of March 1st.

The annual address to the graduates was delivered by Rev. A. G. Haygood, D. D., of Oxford, Ga. (See page 72.) The valedictory was delivered by Dr. Miller B. Hutchins, Lawrenceville, Ga., and was one of the best we ever listened to. The following are the graduates:

A. B. Ashworth, R. H. Bowers, O. C. Brittain, S. W. Brooks, Georgia; C. O. Cargile, Louisiana; W. M. Carter, T. J. Crawford, J. M. Davis, C. A. Davenport, Georgia; T. B. Duckett, South Carolina; D. G. Elder, C. F. Farmer, S. H. Green, R. A. Hardeman, W. P. Harden, B. A. Henry, J. H. Hockenhull, W. H. Hudson, M. B. Hutchins, Georgia; C. B. Jackson, Alabama; H. F. Jackson, J. F. Lacewell, L. L. Lawrence, Georgia; J. H. Maddox, Texas; P. E. Murray, C. E. Murphey, Stephens Neal, M. J. Newberry, B. A. Noland, W. A. Palmour, T. E. Pennington, S. J. Smith, Georgia; C. L. Poole, South Carolina; W. I. M. Smith, Texas; J. M. Sullivan, O. W. Turner, J. D. Veal, G. W. Wallis, Georgia.

The faculty offered three prizes for general proficiency, to be known as the 1st, 2d and 3d honor prizes. These prizes were won respectively by Dr. Miller B. Hutchins, of Georgia; Dr.

W. H. Hudson, of Georgia, and Dr. C. B. Jackson, of Alabama. They consisted of three beautiful gold medals appropriately engraved.

Dr. Calhoun offered an opthalmoscope for the best report of the eye, ear and throat clinic. Won by Dr. C. F. Farmer.

Dr. W. F. Westmoreland offered a prize of a transfusion apparatus for the best report of surgical clinic. Awarded Dr. Stephens Neal.

Dr. Taliaferro a case of obstetrical forceps for the best report of his clinics. Awarded Dr. C. E. Murphey.

Dr. J. S. Todd offered a National Dispensatory for the best paper on materia medica and therapeutics. Won by Dr. W. H. Hudson.

The prizes were delivered by Hon. George Hillyer. He told the young men not to presume because in the first battle they had won, that in life they would be the victor. He said: "You have lingered but a few moments in the vestibule of the temple, whose interior decorations are as yet hid from your sight. You have stood but a moment upon the seashore, and but drops of water have fallen on your feet, while the ocean remains yet away. Strive and struggle on, and always in life be honest, upright and brave, and to you victory will come."

### ITEMS.

DR. J. C. AVARY, of this city, has recently returned from New York, where he spent the winter attending the New York Polyclinic.

DR. J. J. CALDWELL, one of the oldest physicians in this city, died March 12th of apoplexy. He was for a long time a resident of Griffin, Ga. He removed to this place about five years ago and has been in active practice since that time.

AT a recent meeting of the Board of Trustees of the Atlanta Medical College, Dr. R. D. Spalding and Dr. James A. Gray were elected to fill the vacancies caused by the death of Dr. Jos. Thompson and Judge W. W. Clayton. Dr. H. H. Tucker, D. D., was elected President.

DR. VIRGIL O. HARDON has removed his office and residence to No. 68 North Forsyth street, Atlanta, Ga.

ISAAC PHILLIPS, the instrument dealer at 13 N. Broad street, is sole agent for the State for the Home Vapor Bath and Disinfector. They can be seen at his rooms.

PALATABLE COD-LIVER OIL.—Ricklin, in the *Pharm. Zeitschrift fur Russland*, recommends the addition of 4 per cent. of ether to cod-liver oil as a means of overcoming the repugnance to this remedy on the part of many patients. He states that this combination does not tax the digestive organs and will not become rancid.—*Journal de Medecine.*

BURKE, the "Old Book Store man," says: "For two weeks I have received an average of twelve orders each day as the result of an advertisement placed in the March number of THE ATLANTA MEDICAL AND SURGICAL JOURNAL."

COMMENCEMENT OF SOUTHERN MEDICAL COLLEGE.—The commencement exercises of the Southern Medical College were held in DeGive's Opera House on the evening of February 25. There were thirty-four graduates. The annual address to the graduates was delivered by Rev. J. B. Hawthorne, D. D., and the valedictory by Dr. W. W. Burckhalter.

OVARIAN CYST SPONTANEOUSLY EVACUATED THROUGH THE BLADDER.—Perthon reports (*Journal de Medecine*) a case of ovarian cyst which had reached the size of a pregnant womb at term, whose contents were spontaneously evacuated through the bladder. The fluid passed *per urethram* was a neutral liquid containing neither albumen nor sugar, and presenting, under the microscope, granular cells and free granular matter. The patient, who was 45 years of age, made a good recovery; the tumor entirely disappeared, and a year later there was no return of it.

TO DELEGATES TO THE AMERICAN MEDICAL ASSOCIATION.—The rates given to the delegates to the American Medical Association, meeting May 4 in St. Louis, have been fixed by the different railroad committees of the country at one and one-third fares for the round trip. Delegates must pay full fare coming, and will receive, on application from the agent at starting point, a cirtificate which, when signed by the chairman of the local committee of arrangements, will entitle them to the reduced return rate. No reduced return ticket will be issued unless the purchaser can show a certificate issued by the agent from whom he purchased the going ticket, and signed by the chairman of the committee of arrangements.

ALEXANDER'S OPERATION.—At a late meeting of the *Societe' Obstetricale et Gynecologique de Paris*, in a discussion upon Alexander's operation of shortening the round ligaments, M. Pajot made the following timely and sensible remarks: "When an operation is performed, of which death is a possible result, I am the first to approve of it, provided it has for its object the saving of a life absolutely in danger. I can understand how a surgeon may attempt the radical cure of a hernia, since hernia may, at any given moment, become a cause of death. But to-day such operations are resorted to under the pretext of correcting a displacement of the womb. Yet it would probably be impossible to cite a case where a woman died because she had a uterine displacement. It is true that in a pregnant woman retroversion may, if not remedied, produce death. But it is proposed, when the womb is empty, to expose the patient to this grave operation. While I accept extreme measures, undertaken to save a woman who has a uterine tumor, or an ovarian cyst, which may destroy life in a short time, I indignantly protest against dangerous operations undertaken for the relief of conditions which in themselves do not imperil life. Uterine displacements of themselves are insignificant, and produce no symptoms unless associated with uterine catarrh or other lesions of the womb. I believe that if the surgeon would fully and honestly explain to women the dangers of this operation, not one would be found willing to expose herself to so serious risks."—*Archives de Tocologie.*

TRYPSIN AS A SOLVENT OF DIPHTHERITIC MEMBRANE.—Messrs. Fairchild Brothers and Foster have asked us to explain to the profession that, owing to the great cost of this product, and their inability to more than keep pace with the actual demand, they cannot send out samples in the usual manner. Trypsin of their make can, however, be had of the leading dealers, in quarter-ounce, half-ounce and ounce bottles, with full directions. They state that, as a solvent of fibrinous exudate, trypsin is most active in a slightly alkaline medium, and that it may be applied with a brush or in the form of spray. It acts quickly and powerfully, not being, like pepsin, dependent on the interaction of an acid.

ARTIFICIAL FEEDING OF INFANTS.—Miahle, in a memoire to the French Academy in 1845, noted his discovery of animal diastase in the saliva identical in its action upon farinaceous matters with the vegetable diastase in malt, and claimed that for young infants unable to digest starchy food, owing to the failure of the saliva to supply this ferment until the teeth are somewhat developed, the use of the malt diastase with farinaceous mixtures would solve the problem of the artificial feeding of infants. Fifteen years later, Liebig, finding, as the result of his investigations, nothing better than what Miahle had proposed, published his famous formula. But it still remained to perfect from this formula an article that would be available to the use of the mother, requiring of her but little time or skill in its preparation. We believe that in the well known Mellin's Food, the skillful chemist has supplied us a food meeting the highest scientific requirements, and what after all is of far greater importance, a food that in actual use sustains the high claims made in its behalf. We hope and believe that when the merits of this food become more widely known and its use more general that the woful infant mortality, in cities, will greatly decrease. It is of this food that Dr. Fothergill speaks in high commendation. We have tried it thoroughly, and it has done most excellent service.

NOT OUR FAULT.—The *Medical Record* takes the *Florida Medical and Surgical Journal* to task for reproducing one of its editorials, and crediting it to the *Medical Review;* whereupon the *Florida Journal* says: We are always sorry whenever anything appears in the *Journal* that is improperly credited, and hold ourselves *a priori* irresponsible for such mistakes. Only once have we had our attention called to this matter—once by the N. Y. *Medical Record*, which took us to task for an editorial copied from the ATLANTA MEDICAL JOURNAL, in which certain statements were credited to the *Medical Review*, instead of the *Medical Record*. We are very sure our esteemed contemporary, the ATLANTA JOURNAL, must have inadvertently made the *mis*-quotation, but we are equally sure it was not our fault.

We respectfully refer the *Florida Journal* to the January number of THE ATLANTA MEDICAL AND SURGICAL JOURNAL, page 703, where it will be seen that the *Record*, and not the *Review*, is quoted. Whose fault is it?

THE RADICAL TREATMENT OF HERNIAL SACS BY TORSION.—For the past twelve months, Mr. Richard Davy has been availing himself of the mechanically simple method of torsion of the hernial sac, so as to effect its complete obliteration. This plan, he urges, inevitably conducts the process of centripetal occlusion and consolidation to the structural neck of the sac; does away with the necessity of ligatures, obviates wounds of the peritoneal membrane, effectually excludes air, and presents the maximum amount of peritoneal superficies for agglutination and eventual plugging. Of course, no surgical treatment is invariably applicable to operative cases, but it is alleged that a wide field is open for this therapeutic agency in strangulated or non-strangulated herniæ, and opened or unopened sacs. Mr. Davy's fourth successful and successive case (voluminous inguinal left hernia in a woman) is at present convalescent in the Westminster Hospital. —*British Medical Journal, March 13.*

MONTHLY METEOROLOGICAL SUMMARY, ATLANTA, FOR FEBRUARY, 1886.

| | |
|---|---|
| Mean Temperature, | 42.0 |
| Highest Temperature, February 23rd, | 65.3 |
| Lowest Temperature, February 5th, | 8.4 |
| Monthly Range of Temperature, | 56.9 |
| Greatest Daily Range of Temperature, | 27.2 |
| Least Daily Range of Temperature, | 8.4 |
| Mean Daily Range of Temperature, | 19.2 |
| Prevailing Direction of Wind, | N. W. |
| Total Movement of Wind, | 7,800 Miles. |
| Highest Velocity of Wind and Direction, | 32 Miles, N. W. 4 & 19. |
| No. of Days on which .01 inch or more of Rain or Snow fell, | 7 |
| Number of Foggy Days, | None. |
| Number of Clear Days, | 13 |
| Number of Fair Days, | 9 |
| Number of Cloudy Days, | 6 |
| Dates of Frosts { Light, | 2, 7, 8, 17, 18. |
| Dates of Frosts { Killing, | 1, 4, 5, 6, 16, 20, 21, 26, |

WE have received the twelfth annual report of the Superintendent of the Cincinnati Sanitarium for the year ending November 30, 1885. From it we learn that 165 patients were admitte during the year, being the largest number of admissions for any year since the Sanitarium was opened in 1873. One hundred and three patients were discharged cured. This showing—under circumstances adverse to general business prosperity felt throughout the land—argues well for the position occupied by the Sanitarium, in the estimation of the people and the medical profession, to whom it looks for patronage and reputation.

